# Climate Change and Telemedicine: A Prospective View

**DOI:** 10.15171/ijhpm.2020.08

**Published:** 2020-01-25

**Authors:** Sareh Keshvardoost, Reza Dehnavieh, Kambiz Bahaadinibeigy

**Affiliations:** ^1^Medical Informatics Research Center, Institute for Future Studies in Health, Kerman University of Medical Sciences, Kerman, Iran.; ^2^Health Foresight and Innovation Research Center, Institute for Futures Studies in Health, Kerman University of Medical Sciences, Kerman, Iran.; ^3^Modelling in Health Research Center, Institute for Future Studies in Health, Kerman University of Medical Sciences, Kerman, Iran.

## Dear Editor,

There was a question regarding the role of healthcare policy-makers and whether they can help to reduce air pollution by promoting and investing in telemedicine systems.

Global warming has become a global political concern due to excessive greenhouse gas emissions of fossil fuels this leaves undesirable effects on climate conditions, human health, and the world economy.^1^

Due to factors like troubling socio-economic conditions, poor infrastructures, and limited resources to prevent and encounter natural calamities, the vulnerability of developing countries to climate change is significantly higher when compared to developed countries. In fact, reports state that the financial loss resulting from climate change in high-income countries has been 1432 billion dollars, equal to only 0.41 percent of their gross domestic product (GDP), while this number is 21 billion dollars for low-income countries, which constitutes about 1.8% of their GDP.^2^ Also, based on the World Environmental Performance Index, currently developing countries do not have a favorable condition and this is alarming as environmental crisis may worsen in these countries in the near future.^3^

On the other hand, increase in air pollution as a result of global warming reduces labor productivity since the prevalence of diseases like cancer, and cardiovascular and respiratory diseases increase. The economic loss resulting from air pollution in countries with the highest amount of greenhouse gas production is estimated to be more than 4% of GDP.^4^

The transportation industry is one of the main sources of greenhouse gas emission. In 2017, the transportation sector was the main producer of greenhouse gases in America with 29% share of greenhouse gas production of the country.^5^ Similarly in Europe, the transportation sector was announced to be the source of 27% of greenhouse gases and 72% of which was attributed to road transportation.^6^

Therefore, reducing the amount of transportation will consequently decrease pollutants and ultimately air pollution. The healthcare industry has also had a considerable role in air pollution due to the high number of required patient healthcare-related trips that were conducted. As aging is a growing phenomenon and chronic diseases are increasing, more healthcare, inpatient, and rehabilitation services will be required for which more patient and paramedic transportations will be necessary and therefore the healthcare industry will continue to have a major role in producing air pollutants.

In these conditions, using telemedicine technology can be quite beneficial. Telemedicine increases patients access to healthcare services through information and communications technology, prevents unnecessary travelling and referrals, and can be utilized as an effective strategy to reduce the production of air pollutants.^7^ Wootton et al have suggested that asynchronous and synchronous telemedicine systems can reduce patients’ travelling 43% and 70 % respectively.^8^

In this regard, various studies have proven the effectiveness of telemedicine. For instance, in a study conducted in California, more than 19 000 specialized clinical teleconsultations were given to 11 281 patients, which consequently reduced the emission of greenhouse gases by 2000 tons due to less transportations.^9^

In another study, conducted in Canada, a wide range of healthcare services were provided in the form of teleconsultation and as a result road trips were reduced by 757 234 km, and transport vehicle carbon dioxide emissions were reduced by 185 tons.^10^

In one of Portugal’s states, teleconsultations provided in fields of neurology, dermatology, rehabilitation, and general surgery led to a 455-ton reduction in greenhouse gas emissions of vehicles.^11^

The rehabilitation department at the University of Sweden, showed that replacing face-to-face consultation with telemedicine reduced carbon dioxide emission 40 to70 times less.^12^

In conclusion, all countries (especially developing countries which are more vulnerable to climate change) can benefit from the environmental advantages of telemedicine which help reduce the destructive effects of global warming. Therefore, healthcare centers should be informed about the benefits of telemedicine to encourage the implementation and development of this technology.

It is essential however, to consider that the establishment of healthcare centers equipped with telemedicine should be based on the amount of demand for medical services which depends on the prevalence and severity of diseases in that region. Additionally, according to Wotton et al,^8^ healthcare centers with a high average referral rates of patients should be considered for telemedicine services as this will help reduce unnecessary referrals. These healthcare centers will play a great role in encouraging the development of telemedicine centers and reducing greenhouse gas emissions.

Although in previous studies the lack of technological and telecommunication infrastructures in developing countries is a barrier for the implementation of telemedicine systems, many countries like China and India are rapidly benefiting from technological advances and studies have shown that the use of telemedicine is growing in these countries.^13^ It is important to emphasize that Store-and-Forward telemedicine can be implemented even with low bandwidth service availability.

It seems that there are two main challenges prior to the adoption of telemedicine systems: (1) the legal and organizational issues, and (2) the technical and internet-related issues. Appropriate healthcare system policy should be established to overcome these barriers.

If policy-makers address these critical issues, it would be a turning point in healthcare. The need to update and upgrade internet infrastructure through the healthcare sector is another significant point in both developed and developing countries that policy-makers need to take into account.

## Acknowledgements

We are very grateful to “Health Foresight and Innovation Research Center” for their helpful suggestions.

## Ethical issues

Not applicable.

## Competing interests

Authors declare that they have no competing interests.

## Authors’ contributions

KB and SK have contributed to conception and design of the letter. RD has contributed to critical revision of the manuscript. All authors had significant contribution and writing up and finalizing the manuscript.

## Authors’ affiliations

^1^Medical Informatics Research Center, Institute for Future Studies in Health, Kerman University of Medical Sciences, Kerman, Iran. ^2^Health Foresight and Innovation Research Center, Institute for Futures Studies in Health, Kerman University of Medical Sciences, Kerman, Iran. ^3^Modelling in Health Research Center, Institute for Future Studies in Health, Kerman University of Medical Sciences, Kerman, Iran.
